# Exploration of Cadmium Dioxide Nanoparticles on Bioaccumulation, Oxidative Stress, and Carcinogenic Potential in *Oreochromis mossambicus* L.

**DOI:** 10.1155/2020/5407159

**Published:** 2020-07-26

**Authors:** Monera A. Al-Abdan, May N. Bin-Jumah, Saud Alarifi

**Affiliations:** ^1^Department of Biology, College of Science, Princess Nourah Bint Abdulrahman University, Riyadh, Saudi Arabia; ^2^Department of Zoology, College of Science, King Saud University, Riyadh, Saudi Arabia

## Abstract

The field of nanotechnology is rapidly expanding with the advancement of novel nanopesticide and nanofertilizers that have the potential for revolutionizing applications in the agricultural industry. Here, we have done chronic toxicity of cadmium dioxide nanoparticles (CdONPs) on fish *Oreochromis mossambicus* (*O. mossambicus*) using oxidative stress and genotoxic biomarkers. In this current study, the value of LC_50_-96 hr of CdONPs has observed 40 *μ*g/ml for *O. mossambicus.* The three sublethal concentrations, e.g., 4, 10, and 20 *μ*g/ml were selected based on the LC_50_ value. The fishes were treated to the above concentration of CdONPs for 21 days and were harvested at 1, 7, 14, and 21 days for evaluation of clastogenicity, mutagenicity, and genotoxicity of NPs. Generally, significant effects (*p* < 0.01) were observed as a dose and duration of exposure. It was observed that lipid peroxidation (LPO) was increased and glutathione was decreased in both tissues. Micronuclei (MNi) were produced significantly in peripheral blood on 21 days at maximum concentration. A similar trend was seen in the damage of DNA with the same manner in terms of the percentage of tail DNA in the lymphocyte, gills, and kidney cells. This study explored the application oxidative stress, comet assay, and micronucleus assay for in situ aquatic laboratory studies using fish *O. mossambicus* for screening the ecomutagenic and genotoxic potential of environmental pollutants.

## 1. Introduction

Engineered nanoparticles are parts of daily life. The application of heavy metals nanoparticles is increasing all over the world. They are incorporated into a wide array of products including sunscreens, clothing, electronics, paints, and automobiles. The continued development and expansion of industrial applications for nanoparticles ensure that they will enter the aquatic environment through the manufacture, use, and disposal of nanoplastics [1]. The effect of metal nanoparticles is still considered to be a big risk for the health of aquatic animals due to accumulation in different tissues of aquatic animals [2]. Due to anthropogenic activity such as manufacturing, agriculture, sewage, and motor vehicle emissions caused metal pollution in especially developing countries [3], metals are nonbiodegradable. Some researchers documented that heavy metals, e.g., Cd, Cr, Hg, and Fe, affected cellular organelles and enzymes involved in metabolism in aquatic animals [4]. These heavy metals release metal ions and interact with nuclear materials and protein and alter the conformational which initiates apoptosis, carcinogenesis, and modulation of the cell cycle [5]. Many researchers have found the generation of reactive oxygen species as well as oxidative stress works as a key role in mutagenicity and ecotoxicity of metals [6–8]. This is a major risk to aquatic flora and fauna especially to fish, which contribute one of the important sources of protein-rich food for animals and humans. Heavy metallic nanoparticles discharge in the wastewaters from various sources and settle down in the sediments of ponds, lakes, and rivers. The nanoparticles adhere to the surface of plankton and get in the food web. Due to the increase of genotoxic in the aquatic ecosystem, the development of specific biomarkers to find out the genotoxic effects on aquatic organisms has gained importance [9, 10]. However, comet assay is now more important in comparison to other assays due to high sensitivity to find out a little level of DNA damage [11]. The data regarding the oxidative stress and mutagenic and genotoxic nature of CdONPs in aquatic animals is meager, especially the data about the chronic genotoxic effect of CdONPs in *O. mossambicus*. Therefore, the current study investigates the underlying mechanism of mutagenic and genotoxic properties of CdONPs in fish *O. mossambicus*.

## 2. Materials and Methods

### 2.1. Experimental Specimens

The experimental specimen fish *Oreochromis mossambicus* belongs to family Cichlidae, and order Cichliformes were bought from fish markets. The fish had a mean length of 18 ± 2.0 cm and an average weight of 260 ± 10.0 g. The fishes were treated with a potassium permanganate solution (0.05%) for 3 min to escape any dermal infections. The fishes were accustomed for 15 days in the aquarium before CdONP treatments. The fishes were fed goat liver and poultry waste material. Every work as reported by Ali et al. [12] was done to maintain optimal conditions during adaptation.

### 2.2. Chemicals

For this experiment, cadmium dioxide nanoparticles (CdONPs) (average particle size ≥ 100 nm ± 4 nm) were purchased from American Elements 10884 Weyburn Avenue Los Angeles, CA, USA. All other chemicals were purchased from local markets.

### 2.3. Characterization of CdONPs

#### 2.3.1. Transmission Electron Microscopy (TEM)

CdONPs (10 mg) were suspended in Milli-Q water (10 ml). The carbon-coated copper grid was immersed into the suspension (40 *μ*g/ml) of CdONPs, and the grid was dried in the incubator for 24 hr. After drying the grid, the image of nanoparticles was captured by using a transmission electron microscope (JEOL Inc., Tokyo, Japan) at 120 kV. We have captured images of 10 areas of the TEM grid.

#### 2.3.2. Determination of the Hydrodynamic Size of CdONPs

The size and zeta potential of CdONPs in aqueous solution were measured by using dynamical light scattering (DLS, Nano-Zeta Sizer-HT, Malvern, UK) as described by Alarifi and Alkahtani [13]. CdONPs were suspended (40 *μ*g/ml) in double-distilled water. The nanoparticle suspension was sonicated at 40 W for 10 min by a sonicator.

### 2.4. Experimental Design and Evaluation of Sublethal Concentrations

The fishes were put in an experimental glass tank (dimensions 40.30 × 50.10 × 40.30 cm) for 4 days in a semistatic system. Ten fishes were put in each tank. The suspension of CdONPs (10 mg) was prepared in 10 ml ultrapure water and sonicated at 40 mV for 15 minutes using a sonicator (Q-Sonica). The different concentrations of CdONPs (0, 1, 10, 20, 40, 80, and 150 mg/L) were exposed to *Oreochromis mossambicus* for 4 days.

During exposure, fishes were not fed to avoid the adherent of CdONPs to food materials. The experimental water (20 liters) was exchanged every day before the exposure of NPs. After 4 days, the LC_50_ value of CdONPs was determined as 40.0 *μ*g/ml, applying the probit analysis method as reported by Finney [14]. Based on LC_50_ value (4 days), the three test doses of CdONPs, viz., sublethal I (1/10 of LC_50_~4 *μ*g/ml), sublethal II (1/4 of LC_50_~10 *μ*g/ml), and sublethal III (1/2 of LC_50_~20 *μ*g/ml) were determined.

The fish *Oreochromis mossambicus* were treated to the three sublethal doses of CdONPs for 21 days in a semistatic system. The exposure was continued up to 21 days, and tissue sampling was done at intervals of 1, 7, 14, and 21 days at the rate of 5 fishes per duration. An additional set for the positive control (cyclophosphamide 20 mg/kg body weight) was separately maintained.

The fishes were sacrificed on each sampling day, and blood, gills, and kidney tissues were collected for the study of oxidative stress and genotoxicity. The muscle tissue was collected to analyze the bioaccumulation of Cd ^2+^ ion in fish. For histological analysis, the tissues were fixed in Bouin's fixative in small glass tubes. The physicochemical properties of test water, such as temperature, pH, total conductivity, dissolved oxygen, and total hardness, were analyzed by standard methods [15].

### 2.5. Preparation of Sample for ICP-MS

The freeze muscle tissue (5 mg) was mixed with concentrated nitric acid (HNO_3_, 10 ml) and perchloric acid (HClO_4_, 2 ml) in the flask. The flask was heated on a hot plate up to 100°C in fume hood till the yellow color was disappeared. Then, hydrogen peroxide (50 *μ*l) was added. The digested sample was vaporized up to 1.5 ml and diluted with dH_2_O to 50 ml and filtered with a Whatman filter paper. The samples were analyzed using inductively coupled plasma mass spectrometry (ICP-MS) [15].

### 2.6. Oxidative Stress

#### 2.6.1. Preparation of Tissue Lysate

The gills and kidney of CdONPs exposed to fish were washed with cold phosphate-buffered saline and collected in a small tube and minced in small pieces in lysis buffer through the homogenizer and centrifuged at 13000 rpm for 15 min at 4°C, and the supernatant (cell lysate) was put on ice for further tests for reduced glutathione (GSH), lipid peroxide (LPO), catalase (CAT), and glutathione-S-transferase (GST). The quantity of total protein in cell lysate was evaluated by the Bradford method [16] using bovine serum albumin as the standard.

#### 2.6.2. GSH Test

The content of GSH was evaluated according to Ellman's [17] method. 100 *μ*l cell lysate was added with TCA (5%, TCA 900 *μ*l) and centrifuged at 3000 g for 10 min at 4°C. Again, 500 *μ*l supernatant was added with DTNB (0.01%, 1.5 ml), and OD of the mixture was taken at 412 nm. The quantity of glutathione was represented in mU/mg protein.

#### 2.6.3. LPO Test

LPO was evaluated according to Ohkawa et al., [18] method. Cell lysate (100 *μ*l) was added with sodium phosphate buffer (1.9 ml, 0.1 M, pH 7.4) and incubated for 60 min 37°C. Later incubation TCA (5%) was mixed and centrifuged at 3000 g for 10 min at 25°C to collect the supernatant. Then the supernatant was added with 1 ml TBA (1%) and boiled at 100°C for 30 min in a water bath. OD of the mixture was taken at 532 nm and was converted to MDA and expressed in terms of mU/mg protein.

#### 2.6.4. Catalase (CAT)

CAT enzyme was evaluated according to Ali et al. [19].

#### 2.6.5. Glutathione-S-transferase

The activity of glutathione-S-transferase was evaluated by assessing the alteration of absorbance induced by the presence of glutathione dinitrobenzene complex as a product of the reaction between GSH and CDNB [20]. The working solution (0.1 M phosphate buffer, 10 mM GSH, and 60 mM EDTA), CDNB (10 mM), and enzyme source were mixed in a cuvette. The change in absorbance was measured every 30 s for 5 min at 340 nm. The GST level was presented as mU/mg protein.

### 2.7. MN Assay

After sampling the blood, the blood smear was made on the frosted glass slides. The smeared slides were dried at RT and fixed in the method for 10 minutes, and after air drying, the slide was stained with 6% Giemsa in the buffer (pH 6.8) for 30 min. Scoring of micronuclei (MNi) was done using a light microscope (Leitz Wetzlar Germany, 100X with oil). A total of 10000 erythrocytes were examined for each concentration. The micronucleus body was identified according to as described by Ali et al. [12].

MN frequency was calculated as follows:

(1) $$\begin{array}{c} \text{MN}\%=\frac{\text{Number of cells containing micronucleus}}{\text{Total number of cells counted}}\times 100. \end{array}$$

### 2.8. Comet Assay

The DNA damaging potential of CdONPs on different tissues of *O. mossambicus* was assessed using the single-cell gel assay [21]. The gills and kidney tissues (about 50 mg each) were washed two times with chilled phosphate-buffered saline (Ca^2+^ Mg^2+^ free) to remove blood cells and transferred to ice-cold homogenization buffer (1-X Hanks' balanced salt solution, 20 mM EDTA, 10% dimethyl sulphoxide (DMSO), pH 7.0-7.5). The tissue was cut into small pieces using scissors and finally homogenized to obtain a single-cell suspension. The suspension of the cell was centrifuged at 3000 rpm at 4°C for five min, and the cell pellet was finally suspended in chilled phosphate-buffered saline for SCGE assay. Lymphocyte cells were isolated from the blood histopaque density gradient centrifugation method, and the cells were diluted 10-fold for the single-cell gel assay. The two slides were prepared from each fish (25 cells per slide) (250 cells per dose), and the slides were randomly scored using an image analysis system (Komet-5.5, Kinetic Imaging, United Kingdom) attached to a fluorescent microscope (Leica) equipped with appropriate filters. The parameter selected for quantification of DNA damage was percent tail DNA (i.e., %tail DNA = 100 − %head DNA) as determined by the software.

### 2.9. Statistical Analysis

Data were expressed as the mean (±SE) and analyzed by one-way analysis of variance (ANOVA). A *p* value of less than 0.05 and 0.01 was considered statistically significant. Minimum three independent experiments were done in duplicate for each treatment.

## 3. Results

### 3.1. Experimental Water Quality

Table 1 shows the properties of test water quality. During the experiment, the water quality parameters such as pH of water (5.88-7.40), temperature (from 23 to 24.8°C), and dissolved oxygen (DO) (6.32-8.0 mg/l) are found (Table 1). The total hardness of test water was found to be 154.0-190 *μ*g/ml as CaCO_3_. The chloride ion level and conductivity of test water were 45.06 to 53.0 *μ*g/ml and 241.2-289 *μ*M/cm, respectively (Table 1).

### 3.2. Characterization of CdONPs

**Figure 1:**
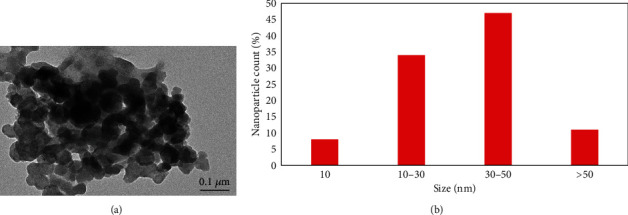
(a) TEM image of CdONPs. (b) Percentage of CdONP size.

The CdONPs have specific properties relative to CdO bulk particle counterparts which impart them beneficial characteristics; they may also bestow them with unique mechanisms of toxicity. We have characterized the size of CdONPs, and their average size was 45.20 ± 3.80 nm (Figures 1(a)–1(b)).  Figure 1(a) shows the TEM image of nanoparticle, and most of the particles are round. The hydrodynamic size of CdONPs was 84.50 nm, and zeta potential was -9.3 mV.

### 3.3. Behavioral Response of Fish and Median Lethal Concentration

**Figure 2:**
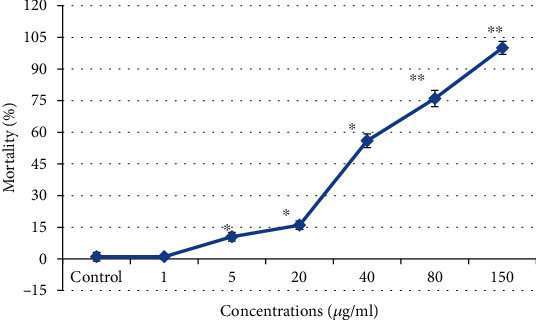
Acute toxicity of CdONPs on fresh water fish *Oreochromis mossambicus.n* = 3; ^∗^*p* < 0.05 and ^∗∗^*p* < 0.01 vs. control.

After exposure to CdONPs (1, 5, 10, 20, 40, 80, and 150 *μ*g/ml), the freshwater fish showed abnormal behavior and we have observed 1%, 10.5%, 14%, 56%, 73%, and 100% mortality in 96 h, respectively (Figure 2). LC_50_ -96 h value with 95% confidence of CdONPs to fish was 40 *μ*g/ml (Figure 2). Behavioral alterations of fish were noticed up to 4 days, and this might be due to intoxication of CdONPs. Due to these toxicities, fish was lost of swimming equilibrium, calmed down to the bottom of the tank, restless, and died without showing any movement and feeding activities at a maximum concentration of CdONPs.

### 3.4. Bioaccumulation of CdONPs

The Cd accumulation in various tissues such as the muscles, gills, and kidney of exposed fish was determined using ICP-MS, and the concentration of Cd metal increases according to the concentration-dependent manner (Table 2). The highest cadmium from CdONPs was found in the gills as compared to the muscles and kidneys (Table 2). The Cd bioaccumulation in different tissues of fish was gills > muscles > kidney (Table 2).

### 3.5. Induction of Oxidative Stress

**Figure 3:**
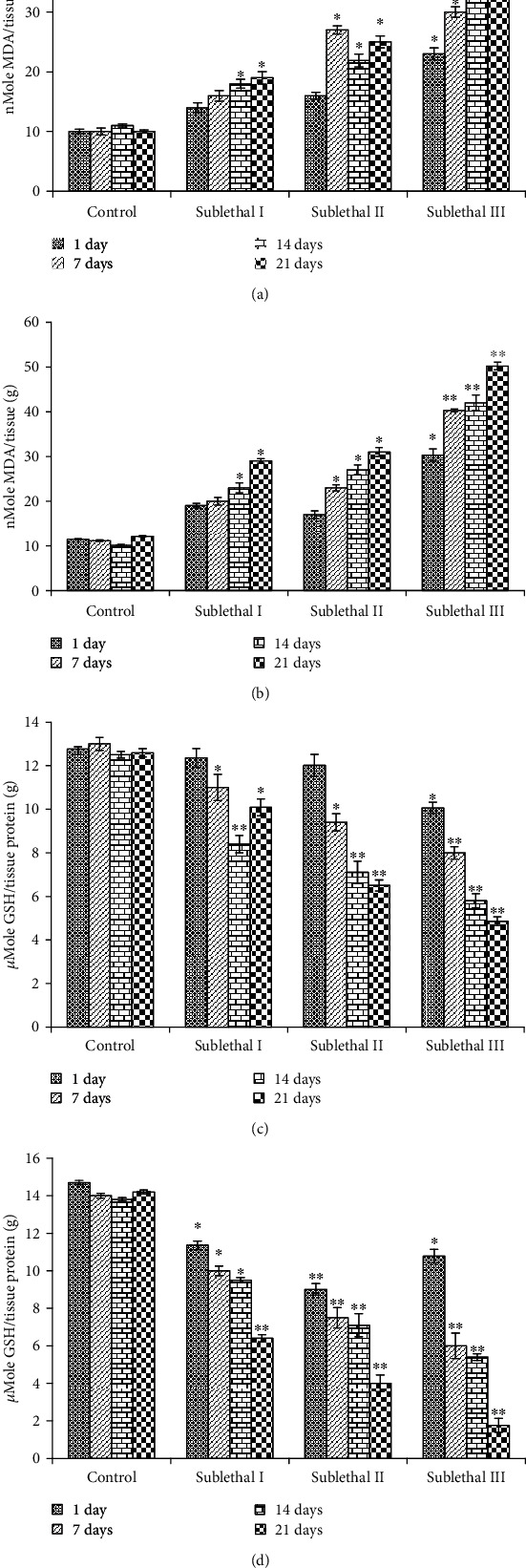
(a) Level of LPO in gill tissue. (b) Level of LPO in kidney tissue. (c) Level of GSH in gill tissue. (d) Level of GSH in kidney tissue. Each value represents the mean ± SE of three experiments. ^∗^*p* < 0.05 and ^∗∗^*p* < 0.01 vs. control.

**Figure 4:**
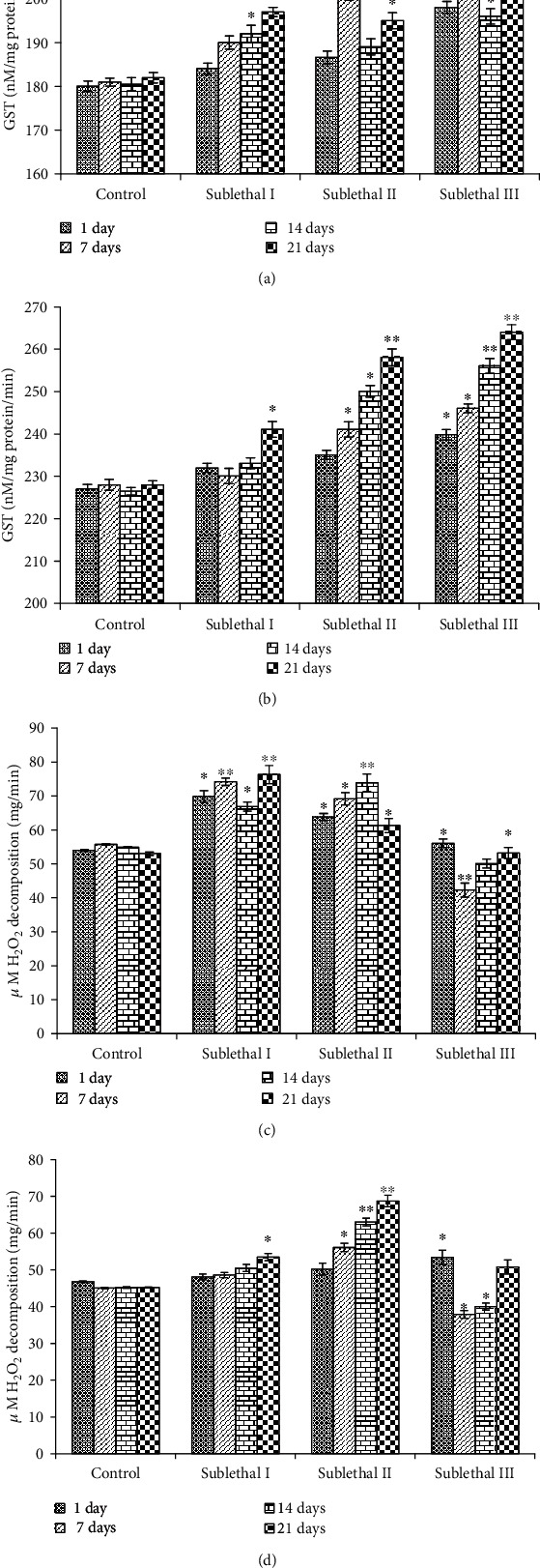
(a) Level of glutathione-S-transferase (GST) in gill tissue. (b) Level of glutathione-S-transferase (GST) in kidney tissue. (c) Level of catalase in gill tissue. (d) Level of catalase in kidney tissue. Each value represents the mean ± SE of three experiments. ^∗^*p* < 0.05 and ^∗∗^*p* < 0.01 vs. control.

The lipid peroxide (LPO) level was measured by measuring the formation of MDA in the gills and kidney tissue of fish. The LPO level was significantly increased at sublethal III dose (21 days) (Figures 3(a) and 3(b)). Exposure of NPs at sublethal I, II, and III for 21 days significantly reduced the GSH level in both tissues of fish (Figures 3(c) and 3(d)). The effect of CdONPs on the GST level in both tissues of fish significantly increased the concentration and time-dependent manner (Figures 4(a) and 4(b)).

Catalase activity in both tissues is significantly induced at sublethal I for the 7^th^ and 21^st^ day, but on the other hand, catalase activity was reduced at sublethal III for the 21^st^ day (Figures 4(c) and 4(d)).

### 3.6. Induction of Micronuclei (MNi)

**Figure 5:**
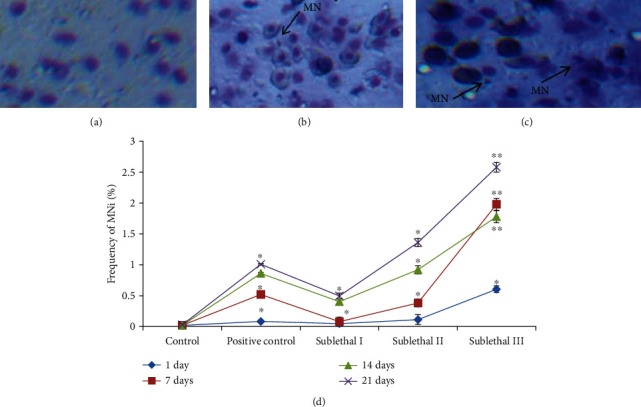
Induction of micronucleus (MN) in erythrocytes of *O. mossambicus* after exposure of different concentrations of CdONPs for 1, 7, 14, and 21 days. (a) Erythrocytes of control *O. mossambicus*. (b) MN in erythrocytes of *O. mossambicus* at sublethal II for 21 days. (c) MN in erythrocytes of *O. mossambicus* at sublethal III for 21 days. (d) Percentage of MN in erythrocytes of *O. mossambicus*. Each value represents the mean ± SE of three experiments. ^∗^*p* < 0.05 and ^∗∗^*p* < 0.01 vs. control.

The mutagenic effect of CdONPs on fish was observed (Figures 5(b)–5(d)). The data showed the significant formation of micronuclei at sublethal II and III CdONPs exposure at the 21^st^ day (Figures 5(b)–5(d)). Also, we have observed that the formation of MNi was in a concentration and time-dependent manner, and on the 21^st^ day, there were 1.5-fold increases from lower to maximum concentrations. The negative control was unable to induce nonsignificant MNi frequency fish.

### 3.7. DNA Damage

**Figure 6:**
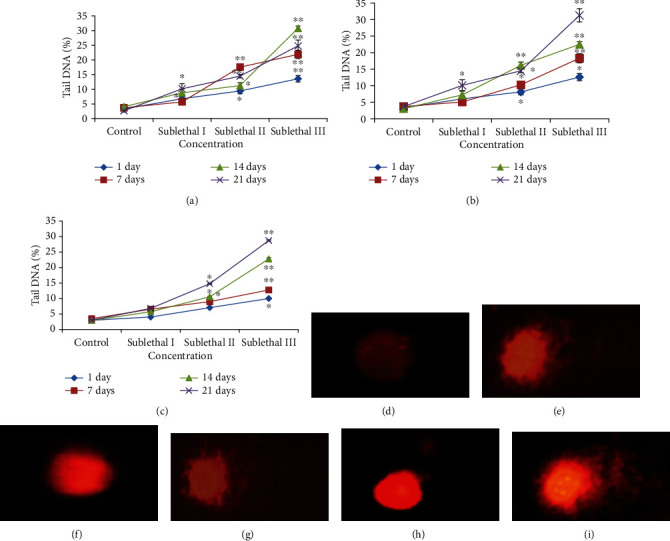
DNA damage in different tissues of *O. mossambicus* after exposure of different concentrations of CdONPs for 1, 7, 14, and 21 days. (a) Tail DNA (%) in lymphocytes. (b) Tail DNA (%) in gill tissue. (c) Tail DNA (%) in kidney tissue. (d) Control lymphocytes. (e) Lymphocyte cells at sublethal III exposure for 21days. (f) Control gill cells. (g) Gill cells at sublethal III exposure for 21 days. (d) Control kidney cells. (g) Kidney cells at sublethal III exposure for 21 days. Each value represents the mean ± SE of three experiments. ^∗^*p* < 0.05 and ^∗∗^*p* < 0.01 vs. control.

The genotoxicity of CdONPs on freshwater fish was determined using single gel electrophoresis and DNA damage as measured in % tail DNA in lymphocyte, gills, and kidney tissue. During electrophoresis, the DNA of all tissues was migrated faster towards the anode at sublethal III concentration than the sublethal I concentration exposure (Figures 6(c)–6(h)). The order of DNA damage in different tissues of fish was gills > lymphocyte > kidney (Figures 6(a) and 6(b)).

## 4. Discussion

Cadmium dioxide nanoparticles (CdONPs) are found naturally in environments and parts of many commercial products, but little is known about their potential hazard on the freshwater organism. Several studies have been carried out about the accumulation of Cd CdONPs [22]. Shaw and Handy [23] reported that copper metal bio accumulated more in the liver in comparison to other organs such as the gills and intestine of fish. The distribution of Cd^2+^ during the study and distribution of CdONPs in *Oreochromis mossambicus* has been observed in the gills than in the kidney. The alteration of behavior is the result of adaptations to the exchange environment. Behavioral changes are a sensitive parameter of an animal's response to stress. Any change in the behavior of freshwater fish indicates a drop in water quality. We have observed the immediate reaction of fish after exposure to the acute concentration of nanoparticles. Fish moved to surface, and erect fins and secreted mucous were seen after exposure to CdONPs. The behavioral changes of this study were following the findings of Khunyakari et al. [24] for *C. carpio* and *Poecilia reticulata* under the effect of copper.

Maintaining the stress to it is the minimum stage, and applying their energy to acclimatize the changing water quality afore for activities may be the reason for behavioral changes in fish under the effect of CdONPs. Bioaccumulation of Cd^2+^ in different tissues after exposure to CdONPs depends upon the physiochemical nature of nanoparticles and water. Nussey et al. [25] have reported that metal (e.g., Cd and Zn) toxicity is affected by the physical-chemical characteristic of test water. The acclimatized dose of Cd was 2 and 0.25 *μ*g/l in surface freshwater bodies in the United States or acute and chronic exposure [26]. In the current study, the physical-chemical characteristics of test water were maintained constant to reduce their effect on metal nanoparticle toxicity. The accumulation of metals indicates pollution of the metal and detects their route of intake and excretion [3, 27]. Some researchers advocated that heavy metals accumulated in tissues such as the gills, kidney, spleen, and liver in chronic exposure [28]. Exposure to heavy metals increases mucus secretion in fish to prevent gill uptake; hence, high levels of metals found in this tissue might be due to mucus-bonded metals. Lipid peroxidation occurred due to the reaction of ROS and membrane lipids [29]. MDA is an important by-product of polyunsaturated fatty acids. Lipid peroxidation by-product MDA level has been measured in the gills and kidney tissues of freshwater fish after exposure to CdONPs. LPO levels were significantly increased among controls and treatment groups in both tissues. Ali et al. [30] have reported that oxidative stress is a major mechanism of ecotoxicity. GSH and LPO (Figure 3) were increased, and GST and catalase (Figure 4) were increased at lower concentrations and increased higher concentration, indicating that toxic effects were related to oxidative stress.

In this study, we have used alkaline single-cell gel electrophoresis to detect the genotoxic potential of CdONPs in different tissues of freshwater fish because at higher pH > 13, the expression of alkali labile sites and single-strand breaks was increased. We have seen that fragments of DNA and loosed DNA loop were migrated towards the anode and forming the tail of a comet as seen in Figure 6. The percentage of tail DNA is an important parameter to express the genotoxicity.

On the basis of the current study, the bioaccumulation of Cd ion was more in the muscle tissue than in the gills and kidney tissues after exposure to CdONPs for the 21^st^ day. However, the genotoxicity effects of CdONPs were more in lymphocyte cells in comparison to the gill and kidney. This can be confirmed that CdONPs were eco-genotoxic and carcinogenic to aquatic organisms.

## Figures and Tables

**Table 1:** Physiochemical characteristics of test water.

Parameters	Values
Temperature	23-24.8°C
pH	5.88-7.40
Dissolved oxygen (mg/l)	6.32-8.00
Total hardness (as CaCo3) (*μ*g/ml)	154.0-190
Chloride (*μ*g/ml)	45.06-53.0
Conductivity (*μ*M/cm)	241.2-289

**Table 2:** Bioaccumulation of Cd in gill, kidney, and muscles of fish.

Tissues	Control	Exposure concentrations		
		Sublethal I	Sublethal II	Sublethal III
Gill	0.062 ± 0.0 13	0.09 ± 0.0 1	0.102 ± 0.0 1	0.119 ± 0.01
Kidney	0.065 ± 0.012	0.063 ± 0.03	0.087 ± 0.016	0.098 ± 0.02
Muscles	0.088 ± 0.002	0.109 ± 0.002	0.116 ± 0.002	0.124 ± 0.001

All values are the mean ± SE.

## Data Availability

All data generated or analyzed during this study are included in the article.
